# The efficacy of pharmacological interventions to improve cognitive and behavior symptoms in people with dementia: A systematic review and meta‐analysis

**DOI:** 10.1002/hsr2.913

**Published:** 2022-11-06

**Authors:** Wisdom K. Takramah, Livingstone Asem

**Affiliations:** ^1^ Department of Epidemiology and Biostatistics, School of Public Health University of Health and Allied Sciences Ho Ghana; ^2^ Department of Biostatistics, School of Public Health University of Ghana Accra Ghana; ^3^ Department of Health Policy, Planning and Management, School of Public Health University of Health and Allied Sciences Ho Ghana

**Keywords:** aging, behavioral symptoms, cholinesterase inhibitors, cognition, dementia, memantine

## Abstract

**Background and Aims:**

Dementia is becoming a major global public health menace in the aging population affecting 47 million people globally. Dementia has no cure and effective interventions. Treatment of dementia is a big problem. The most common symptomatic medications for cognition, behavior, and global functioning among patients with dementia currently are cholinesterase inhibitors and memantine. However, Information on the effectiveness of cholinesterase inhibitors for dementia is conflicting and controversial. Thus, this makes it difficult for decision‐makers, healthcare providers, patients, and caregivers to decide on the most effective intervention. The current meta‐analysis sought to investigate the efficacy of pharmacologic interventions to improve cognitive and behavioral symptoms in people with living dementia.

**Methods:**

This current systematic review and meta‐analysis used the preferred reporting items for systematic reviews and meta‐analyses to ensure accuracy and comprehensiveness. The Cochrane MEDLINE, Database of Systematic Reviews, and other databases were thoroughly searched for relevant studies. We selected Studies such as randomized controlled trials published in English with a sample size of at least 20 subjects. We selected and applied the random‐effects meta‐analysis as the most preferred model because of the heterogeneity across studies. The computation of the weighted effect size was based on the result from the test of heterogeneity.

**Results:**

Twenty‐two studies were finally used in the meta‐analysis. The study subjects who received donepezil 5 mg/day, donepezil 10 mg/day, and galantamine 24 mg/day had improved cognition symptoms (ADAS‐cog) score of −1.46 (95% CI = −2.24, −0.68, *z* = 3.67, *p* < 0.001), −2.31 (95% CI = −3.30, −1.31, *z* = 5.45, *p* < 0.001) and −3.04 (95% CI = −4.16, −1.92, *z* = 5.31, *p* < 0.001) respectively.

**Conclusion:**

The current meta‐analysis suggests significant benefits of cholinesterase inhibitors such as donepezil (5 and 10 mg/day) and galantamine on cognitive symptoms.

## 1 INTRODUCTION

Dementia is a debilitating disease and a public health problem of concern. It is prevalent in the aging population. Dementia is one of the most common forms of degenerative health problems among the aging population and poses a serious health burden with high socioeconomic costs.^1^ The global prevalence of dementia in 2018 was 50 million people. Hence, this was estimated to surge up to 82 million by 2030 and triple to 152 million people by 2050.^2^ There is no treatment or effective intervention for Alzheimer's disease (AD) and it is the most common type of dementia and constitutes 60%–70% of cases.^3^, ^4^ Unfortunately, by the time patients develop dementia, the brain has already suffered serious damage and severely impacts cognition and autonomy.^5^ To this end, stringent efforts to develop and implement interventions to manage and mitigate the progress and risks of dementia cannot be over‐emphasized. According to Dou et al.,^6^ the key role medications or interventions play in the management and treatment of dementia, and finding solutions to problems with depression, behavior and cognition cannot be overemphasized.

The treatment of dementia is a major problem. Although numerous clinical trials have been carried out over the years, there is no effective treatment or cure for dementia.^4^ It is worthwhile to mention that optimal treatment to avert or delay mild cognitive impairment (MCI), cognitive decline, or dementia is uncertain. Even though pharmacological and nonpharmacological interventions have been introduced, as a remedy for dementia, evidence regarding their safety and efficacy is conflicting and confusing. Pharmacotherapy is often the major intervention implemented to improve symptoms or delay the progression of dementia syndromes. The available agents vary concerning their therapeutic actions and are supported by different levels of evidence for efficacy.

One way to address the menace of AD is to identify and block the candidate enzyme from interrupting the neurotransmitter acetylcholine to increase the performance of cholinergic neurotransmission in the brain. A family of medicines such as cholinesterase inhibitors was developed to inhibit the activities of the candidate enzyme.^7^ Thus, pharmacological interventions such as cholinesterase inhibitors and memantine are the only currently available symptomatic medications for cognition, behavior, and global functioning in patients with dementia.^8^ Internationally recognized and accepted cholinesterase inhibitors for treating dementia include donepezil, rivastigmine, and galantamine. They are considered symptomatic therapies and are not believed to be neuroprotective or alter the underlying disease trajectory.^8^ Cholinesterase inhibitors offer some relief from the symptoms of AD for some people for a limited period. Cholinesterase inhibitors are identified as very effective in treating AD, not only in the mild‐to‐moderate stage but also when symptoms become more severe.^6^ US Food and Drug Administration (ADA) has approved cholinesterase inhibitors for the symptomatic management and treatment of dementia for AD.^9^

Pharmacological interventions such as donepezil, galantamine, and rivastigmine have been tested in patients with AD and in patients with vascular dementia, dementia with Lewy bodies, dementia associated with Parkinson's disease, and MCI in the last decade,.^10^ Even though available information on the effectiveness of these medicines is questionable, the results are often presented in such a way as to create a false perception of efficacy.

Many factors and their interactions might have contributed to the cholinesterase inhibitors tested in primary RCTs not achieving their intended significant therapeutic effects. Factors such as flaws in the design, sample size, inappropriate analysis, and some uncontrolled characteristics of the subjects could grossly affect the quality of the study results. Thus, identifying these extraneous factors in meta‐analysis will go a long way to help improve the quality of future clinical trials on a similar topic area. Information on the effectiveness of cholinesterase inhibitors for dementia is conflicting and controversial. This makes it difficult for decision‐makers, healthcare providers, patients, and caregivers to decide on the most effective intervention. Thus, this meta‐analysis sought to thoroughly conduct a meta‐analysis to investigate the effectiveness of pharmacologic interventions to improve cognitive and behavioral symptoms in people with dementia.

## 2 METHODS

### 2.1 Protocol and registration

The protocol was registered and published with PROSPERO (registration: CRD42020159408) in accordance with the criteria in the preferred reporting items for systematic reviews and meta‐analyses (PRISMA).^11^, ^12^

### 2.2 Types of studies

Studies that included all randomized controlled trials (RCTs), randomizing either individuals or clusters and open‐label studies, investigating the effects of pharmacological interventions on cognitive and behavioral symptoms in people with dementia met the inclusion criteria. Studies that have a cognitive symptoms‐related outcome measure as a primary outcome were also included. Retrospective, cross‐sectional, cohort studies, and quasi‐experimental were excluded.

### 2.3 Types of participants

Those who were diagnosed of dementia, regardless of type of dementia, setting, age, and the severity of cognitive impairment, were included in the study. Studies where dementia was diagnosed using standard medical or research diagnostic criteria to rule out other conditions and well‐documented descriptions of the methods used for assessment were included.

### 2.4 Types of interventions

The current study included Pharmacological interventions aiming to improve cognitive and behavioral symptoms in people with dementia. Pharmacological interventions used in this meta‐analysis included donepezil (5 and 10 g) and galantamine (24 g). We excluded studies where patients received non‐cholinesterase inhibitors and nonpharmacological interventions such as cognitive training, music therapy, aromatherapy, multisensory stimulation, massage, and animal therapy.

### 2.5 Control

Comparator interventions included placebo and standard treatment.

### 2.6 Outcome measures

Many assessment scales have been developed over the years for use in dementia research and care. However, assessment scales in the areas of behavior and cognition were reviewed in this meta‐analysis. AD Assessment Scale‐Cognitive (ADAS‐cog) and mini‐mental state examination (MMSE) were used to measure cognitive symptoms, and neuropsychiatric inventory (NPI) was used to measure behavioral symptoms.

### 2.7 Cognition

#### 2.7.1 ADAS‐cog

The ADAS‐cog Subscale test is one of the most widely used tools to quantify cognition in medical research studies and clinical trials for new drugs and other interventions. It's more comprehensive than the Mini‐Mental State Exam, and it primarily quantifies language, orientation, and memory. The ADAS‐Cog composes of 11 parts and takes nearly 30 min to complete. Most current studies employ the ADAS‐Cog to quantify cognitive ability.^13^

#### 2.7.2 MMSE

The MMSE is commonly employed to examine cognitive function among the aged population. The medical or clinical researchers with a little training can use the MMSE scale. It takes about 10 min to complete and it measures cognitive functions such as memory, orientation, visual construction attention, X language, and orientation. The score of MMSE scale ranges from 0 to 30 points, and cut‐offs of 23/24 have been used to depict significant cognitive impairment.^14^

### 2.8 Behavior

#### 2.8.1 NPI

The NPI evaluates a wide range of behaviors seen in dementia for both severity and frequency. These include agitation, irritability, delusions, apathy, and depression. The clinician usually administer the ***NPI*** scale in 10 min to a career. The ***NPI*** scale has good psychometric properties and is commonly employed in drug trials, and is concise enough (especially with patients without a wide range of behavioral issues) to consider for use in clinical practice.^15^

### 2.9 Search strategy, trials selection, and data retrieval

This study used the PRISMA to ensure accuracy and comprehensiveness.^11^, ^12^ We searched electronic databases including MEDLINE, Embase, Google Scholar, JSTOR, MODEM, and The Cochrane Database. We also searched the Cochrane Controlled Trials Register for studies investigating the effectiveness of donepezil and galantamine for dementia using the MeSH terms such as dementia, vascular dementia. AD, pharmacological interventions, cholinesterase inhibitors, donepezil, and galantamine.

We included Randomized placebo‐controlled trials and open‐label non‐randomized trials. The study excluded case‐control and cohort studies, dissertations, editorials, and case reports. Trials with a duration of at least 12 weeks and outcome measures such as cognitive and behavioral symptoms met the inclusion criteria. Patients with dementia who received pharmacological interventions including donepezil (5 and 10 gm), galantamine (24 g), and placebo or comparator RCTs that included quantitative outcome measures of cognitive symptoms (MSME and ADAS‐cog) and behavioral symptoms (NPI) were the focus of this study. Two research assistants were tasked to independently assess the relevancy of search results and extract the data into an electronic template designed for this current study. Any disagreements regarding the inclusion of studies were resolved in a consensus meeting. A third reviewer made the definitive decision for study eligibility and data extraction in case of persistent disagreement. We limited the search to articles written and published in English.

### 2.10 Searching other resources

We screened reference lists of included trials and the bibliography of recent meta‐analysis and systematic reviews and relevant recent guidelines. We obtained from the Authors and experts in the field additional published and unpublished randomized trial reports that could not be identified by the search.

### 2.11 Data extraction and analysis

Two trained research assistants independently assessed the relevancy of search results and extracted the data from studies that met the inclusion criteria. Data extracted from each study included detailed characteristics of the trials (settings, outcomes of interest), design features (delivery format, blinding), participant characteristics (diagnoses, age, gender, weight), elements of the experimental and control interventions (frequency, duration, key intervention features). The research assistants obtained information about key variables of interest for investigating the effect moderators such as type of control, sample, country, year of publication, length of follow‐up, number of participants in each group. For each outcome of interest, the change in the mean scores from the baseline and standard deviations on relevant measures from all available evaluations were extracted. Intent to treat (ITT) results were recorded, and if not available, then observed case or per‐protocol outcomes were extracted.

### 2.12 Assessment of risk of bias in included studies

The methodological quality of the relevant studies eligible for inclusion was assessed by two research assistants independently using a modified version of the Scottish Intercollegiate Guidelines Network (SIGN) 50 checklist.^16^ SIGN 50 is a validated quality checklist composed of 10 items and encompasses the most important sources of bias and variation observed in randomized clinical trials. All 10 items were considered in this study. Each item was scored as very good (2), good (1), or poor (0). The data collected on these items were entered into Microsoft Excel and transferred to STATA version 15 for data management and further analysis. The amount of agreement between the rating of each research assistant and the consensus rating was explored. Additionally, interobserver variability by calculating the kappa statistic was examined.

### 2.13 Statistical analysis

The data were extracted and entered into the EXCEL template designed for the purpose of this study and further transferred to STATA version 15 and Review Manager (RevMan) version 5.3 for cleaning and analysis. Descriptive statistics were used to summarize the main study characteristics and the risk of bias. A fixed‐effect model and random‐effect model are the two main types of models that were used to perform a meta‐analysis. To decide on which model was the most plausible, a statistical test of heterogeneity across studies in the meta‐analysis was examined using *I* ^2^ statistics (the percentage of variation across studies that is due to between‐study heterogeneity rather than chance). *I* ^2^ of at least 50% were taken as indicators of heterogeneity of outcomes. Thus, random‐effects meta‐analysis was selected over fixed‐effect meta‐analysis because of the methodological heterogeneity across studies. Effect sizes for continuous data were calculated as the standardized mean difference (SMD) (Cohen's *d*) between treatment and control groups. Assessment scales for cognition (ADAS‐cog and MMSE) and behavior (NPI) were used to collect continuous data and analysis of the weighted mean difference was conducted. The weighted mean difference reflects the difference in change from baseline to endpoint for active treatments (donepezil and galantamine) and control or comparator group, weighted by the inverse variance.^13^ To pool data from studies using different measurement scales for the same outcome, SMD were pooled for continuous outcomes. A *p*‐value < 0.05 was considered for statistical significance.

### 2.14 Model formulation

#### 2.14.1 Fixed‐effect model

In a fixed‐effect model, the usual estimate of a mean effect size consists of weighting every effect estimate, *T*_*i*,_ by its inverse variance, *w*_*i*_. The weight assigned to each study is given as *w*_*i*_ = $\frac{1}{V_{i}}$, where $V_{i}$ is the within‐study variance for study (i).

The weighted mean effect is then computed using the equation:

(1) $$\bar{T.}=\frac{\sum_{i=1}^{k}w_{i}T_{i}}{\sum_{i=1}^{k}w_{i}},$$

that is, the sum of the products *w*_*i*_ *T*_*i*_ (effect size multiplied by weight) divided by the sum of the weights, where *T*_*i*_ is the observed effect size in study (i).

The variance of the combined effect is the reciprocal of the sum of the weights which is given by

(2) $$v.=\frac{1}{\sum_{i=1}^{k}w_{i}}.$$

The standard error (SE) of the combined effect is the square root of the variance,

(3) $$\text{SE}(\overset{®}{T.})=\sqrt{v.}$$

#### 2.14.2 Random‐effect modeling

Thus, total variance in random‐effect model = within‐studies variance + between‐studies variance (denoted by $\tau^{2})$. This is expressed mathematically as $V_{i}^{*}=v_{i}+\tau^{2}$. An estimator based on the method of moments proposed by DerSimonian and Laird for between‐study variance (τ^2^) is defined as

(4) $$\tau^{2}=\frac{Q-(k-1)}{C}=\frac{Q-\mathrm{df}}{C},$$

where *Q* in the equation is Cochrane's *Qtest* which represents total variance and *k* is the number of studies.

If $\tau^{2}$ is negative then it is set to zero because the variance between‐studies cannot be negative where *C* in Equation (4) is a constant and is defined as

(5) $$c=\sum w_{i}-\frac{\sum w_{i}^{2}}{\sum w_{i}}.$$

Now the weight assigned to each study under random‐effect model is given by

(6) $$w_{i}^{*}=\frac{1}{v_{i}^{*}}.$$

The weighted average therefore is computed as

(7) $${\bar{T}}_{.}^{*}=\frac{\sum_{i=1}^{k}w_{i}^{*}T_{i}}{\sum_{i=1}^{k}w_{i}^{*}},$$

that is, the sum of the products $w_{i}^{*}T_{i}$ (effect size multiplied by weight) divided by the sum of the weights, where *T*_*i*_ is the observed effect size in study (i).

The variance of the combined effect is the reciprocal of the sum of the weights under the random‐effect model is defined as

(8) $$v_{.}^{*}=\frac{1}{\sum_{i=1}^{k}w_{i}^{*}}.$$

The standard error of the average effect size is the squared root of the variance defined as

(9) $$\mathrm{SE}\;({\bar{T}}_{.}^{*})=\sqrt{v_{.}^{*}}.$$

## 3 RESULTS

### 3.1 Identification and characteristics of included studies

**Figure 1:**
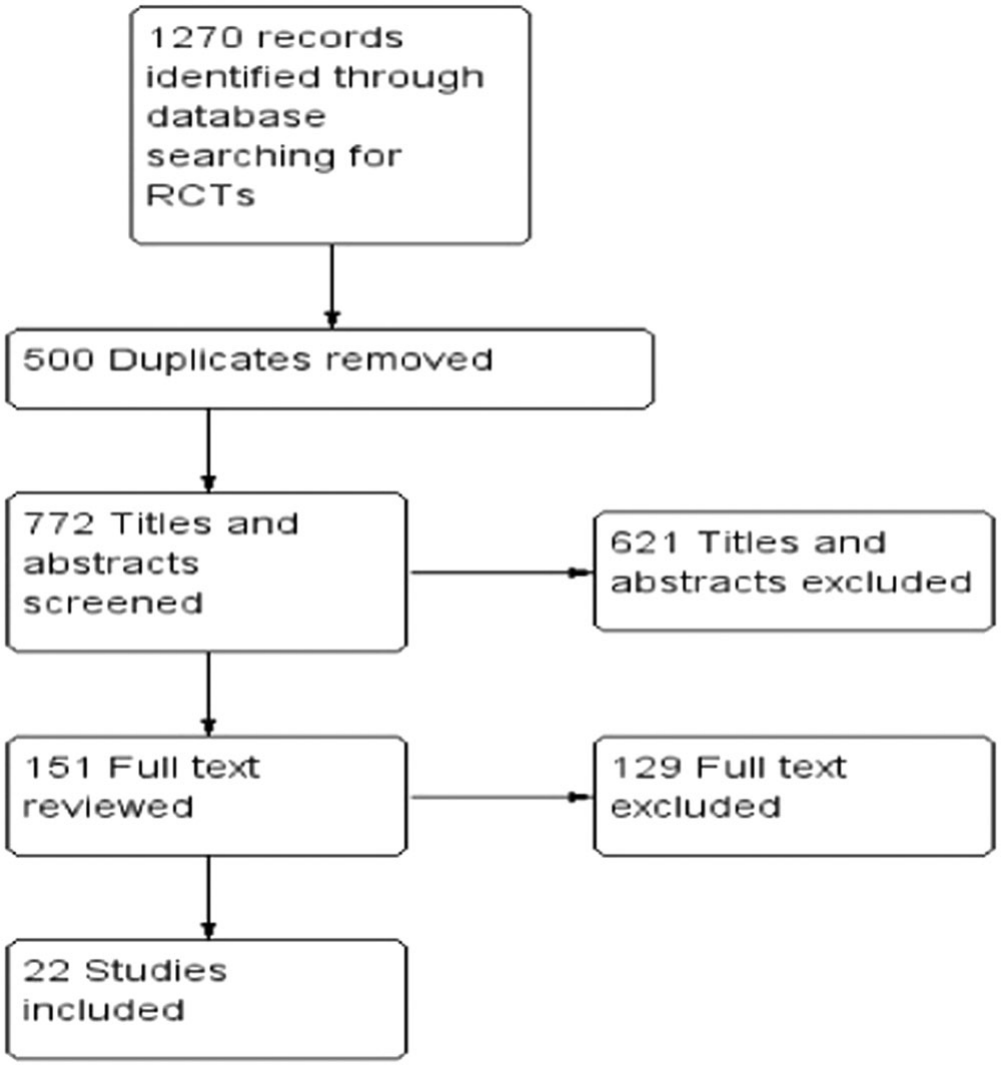
Summary of study selection

We initially identified 1270 studies through database search and screening of the reference lists of previous meta‐analyses. We selected 151 studies for full‐text review shown in the PRISMA diagram (Figure 1). Of the total number of studies initially identified, we removed 500 studies because of duplication. The study excluded 621 titles and abstracts and 129 full‐text studies from the meta‐analysis for reasons like patients were given non‐cholinesterase inhibitors, sample size less than 20, cross‐sectional surveys, and case‐control were used; qualitative outcomes were measured; and studies were not relevant. We finally included twenty‐two^17^ studies in the meta‐analysis. The included studies were 21 randomized placebo‐controlled trials and one open‐label extension study, where 11 studies evaluated donepezil (5 and 10 mg/day) and 11 studies investigated the efficacy of galantamine 24 mg/day (Table 1). The duration of follow‐up ranged from 12 to 96 weeks. The mean age ranged from 71.9 to 85.7 years.

**Table 1:** Summary of basic characteristics of the selected studies for meta‐analysis

Study	Study type	Intervention	Number of patients	Mean (SD) Age (years)	Male (%)	Female (%)	Length of follow‐up time (weeks)
Ikeda et al^18^	RCT	Donepezil 5 mg/day	45	78.8 (5.1)	44.4	55.6	12
		Donepezil 10 mg/day	49	77.7 (6.8)	42.9	57.1	
		Placebo	44	77.2 (6.1)	38.6	61.4	
Mori^19^	RCT	Donepezil 5 mg/day	32	77.9 (6.8)	50.0	50.0	12
		Donepezil 10 mg/day	36	78.6 (6.1)	11.1	88.9	
		Placebo	31	78.6 (4.7)	28.1	71.9	
Dubois^20^	RCT	Donepezil 5 mg/day	183	72.0 (6.8)	65.0	35.0	24
		Donepezil 10 mg/day	173	70.8 (7.5)	75.0	25.0	
		Placebo	170	72.9 (6.5)	65.0	35.0	
Black^21^	RCT	Donepezil 5 mg/day	198	73.7 (0.6)	56.1	43.9	24
		Donepezil 10 mg/day	206	73.9 (0.6)	51.9	48.1	
		Placebo	199	74.2 (0.6)	57.8	42.2	
Rogers^17^	RCT	Donepezil 5 mg/day	153	73.8 (0.67)	31.0	69.0	24
		Donepezil 10 mg/day	150	73.4 (0.65)	39.0	61.0	
		Placebo	154	74.0 (0.65)	39.0	61.0	
Gustavo^22^	RCT	Donepezil 5 mg/day	399	74.2 (0.4)	59.4	40.6	24
		Donepezil 10 mg/day	398	74.8 (0.4)	57.2	42.8	
		Placebo	383	74.3 (0.4)	56.1	43.9	
Friedhoff	RCT	Donepezil 5 mg/day	156	72.9 (0.6)	37.0	63.0	12
		Donepezil 10 mg/day	155	74.6 (0.6)	38.0	62.0	
		Placebo	150	72.6 (0.6)	39.0	61.0	
Gustavo^23^	RCT	Donepezil 5 gm/day	648	73.4 (0.4)	61.4	38.6	24
		Placebo	326	72.3 (0.5)	54.0	46.0	
Ikeda^24^	Open‐label	Donepezil 5 mg/day	26	78.7 (6.6)	50.0	50.0	52
		Donepezil 10 mg/day	21	78.2 (6.6)	4.3	95.7	
		Placebo	28	79.0 (4.6)	32.1	67.9	
Wilkinson^25^	RCT	Donepezil 5 mg/day	168	74.7 (0.6)	62.5	37.5	24
		Donepezil 10 mg/day	162	75.7 (0.6)	62.3	37.7	
		Placebo	161	74.4 (0.6)	54.4	45.6	
Erkinjuntti^26^	RCT	Galantamine 24 mg/day	295	74.9 (0.41)	52.5	47.5	48
		Placebo	164	75.6 (0.56)	51.8	48.2	
Erkinjuntti^27^	RCT	Galantamine 24 mg/day	396	75.0 (6.84)	48.0	52.0	24
		Placebo	196	75.2 (7.32)	46.0	54.0	
Chu^28^	open‐label	Galantamine	32	74.48 (1.61)	28.3	71.7	96
		Historical control group	19	78.89 (1.41)	26.3	73.7	
Tariot^29^	RCT	Galantamine 24 mg/day	253	77.7 (0.4)	33.0	67.0	17
		Placebo	255	77.1 (0.5)	37.8	62.2	
Rockwood^30^	RCT	Galantamine 24 mg/day	239	75.2 (0.45)	46.4	53.6	12
		Placebo	123	74.6 (0.68)	43.3	56.7	
Harandi^31^	RCT	Galantamine 24 mg/day	66	72.5 (5.2)	56.1	43.9	64
		MLC601	66	71.8 (5.7)	56.1	43.9	
Auchus^32^	RCT	Galantamine 24 mg/day	363	72.3 (9.0)	62.0	38.0	26
		Placebo	372	72.2 (8.8)	66.0	34.0	
Suh^33^	Community‐controlled	Galantamine 24 mg/day	80	73.8 (0.8)	77.5	22.5	16
		Placebo	76	76.8 (1.2)	75.8	24.8	
Kurz^34^	Open‐label	Galantamine 24 mg/day	150	76.2 (7.09)	53.4	46.6	96
		Community control	76	76.8 (7.42)	60.0	40.0	
Wilcock^35^	RCT	Galantamine 24 mg/day	220	71.9 (8.3)	36.8	63.2	34
		Placebo	215	72.7 (7.6)	38.6	61.4	
Hager^36^	RCT	Galantamine 24 mg/day	1024	73.0 (8.9)	34.5	65.5	96
		Placebo	1021	73.0 (8.7)	64.1	35.9	

### 3.2 Effectiveness of cholinesterase inhibitors to improve behavior symptoms in people with dementia as measured by NPI

The effect sizes or values less than 0 in the forest plot displayed in Figure 1 shows better results.

**Figure 2:**
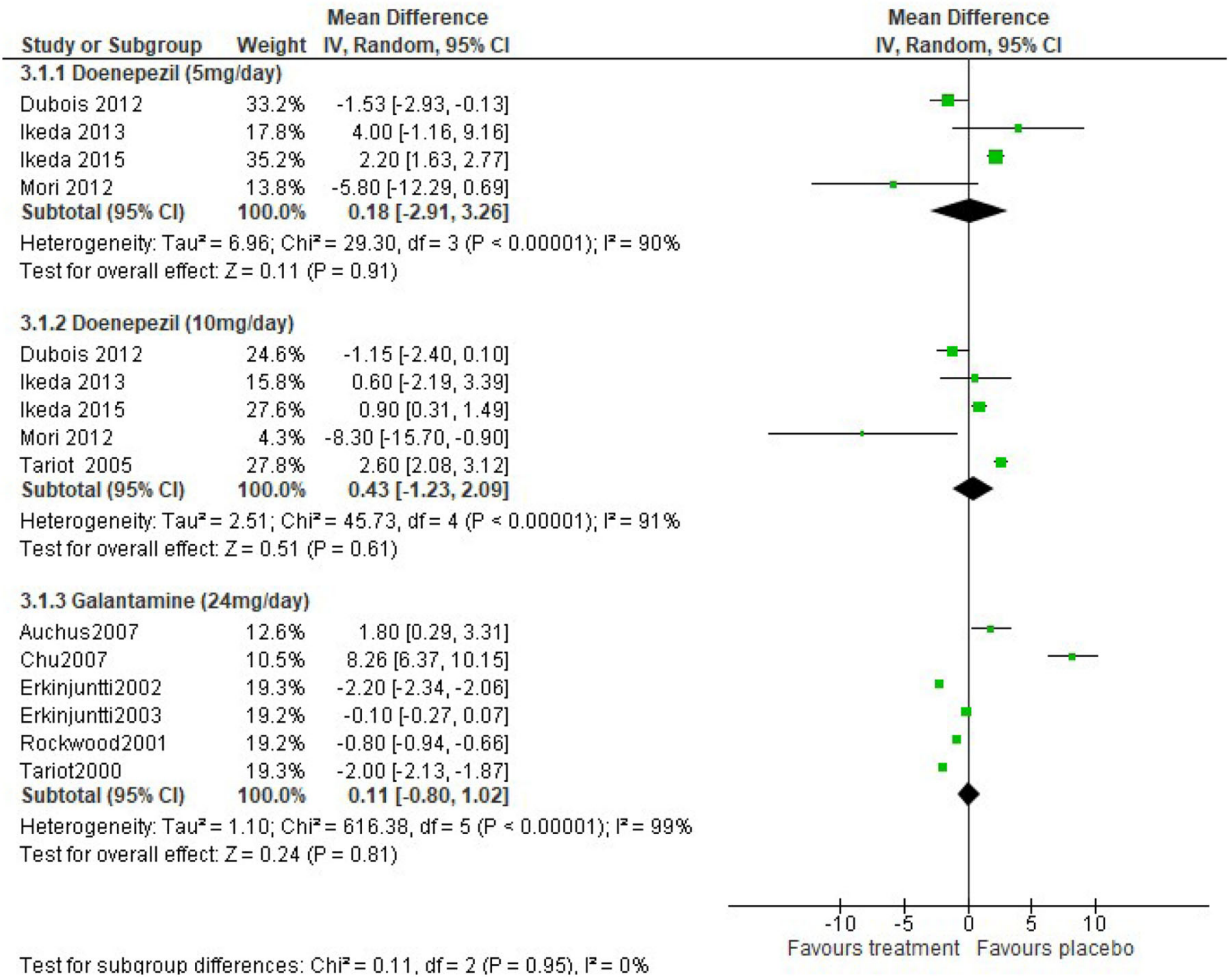
Forest plot of treatment effect of cholinesterase inhibitors on changes in behavior symptoms as measured by neuropsychiatric inventory

In Figure 2, four studies were used in the meta‐analysis to investigate the efficacy of donepezil 5 mg/day to improve behavioral symptoms in people with dementia, five studies were used for donepezil 10 mg/day, while six studies were used for galantamine 24 mg/day.

Generally, there is evidence of heterogeneity or effect size is different across the studies for the donepezil 5 mg/day (Cochran *Q* = 29.30, *p* < 0.001, *I* ^2^ statistic = 90%), donepezil 10 mg/day (Cochran *Q* = 45.73, *p* < 0.001, *I* ^2^ statistic = 91%) and galantamine 24 mg/day (Cochran *Q* = 616.38, *p* < 0.001, *I* ^2^ statistic = 99%). The heterogeneity observed across the studies shows conflicting results for the individual studies. There is no evidence to conclude that the weighted or combined difference in the mean change of NPI score for cholinesterase inhibitors are effective in improving behavior symptoms in people with probable dementia. A behavior symptom of 0.18 (95% CI = −2.91, 3.26, *z* = 0.11, *p* = 0.91, *α* = 0.05), 0.43 (95% CI = −1.23, 2.09, *z* = 0.51, *p* = 0.61, *α* = 0.05) and 0.11 (95% CI = −0.80, 1.02, *z* = 0.24, *p* = 0.81, *α* = 0.05) was recorded for patients treated with donepezil 5 mg/day, donepezil 10 mg/day, and galantamine 24 mg/day, respectively.

### 3.3 Effectiveness of cholinesterase inhibitors to improve cognitive symptoms in people with dementia as measured by ADAS‐cog

**Figure 3:**
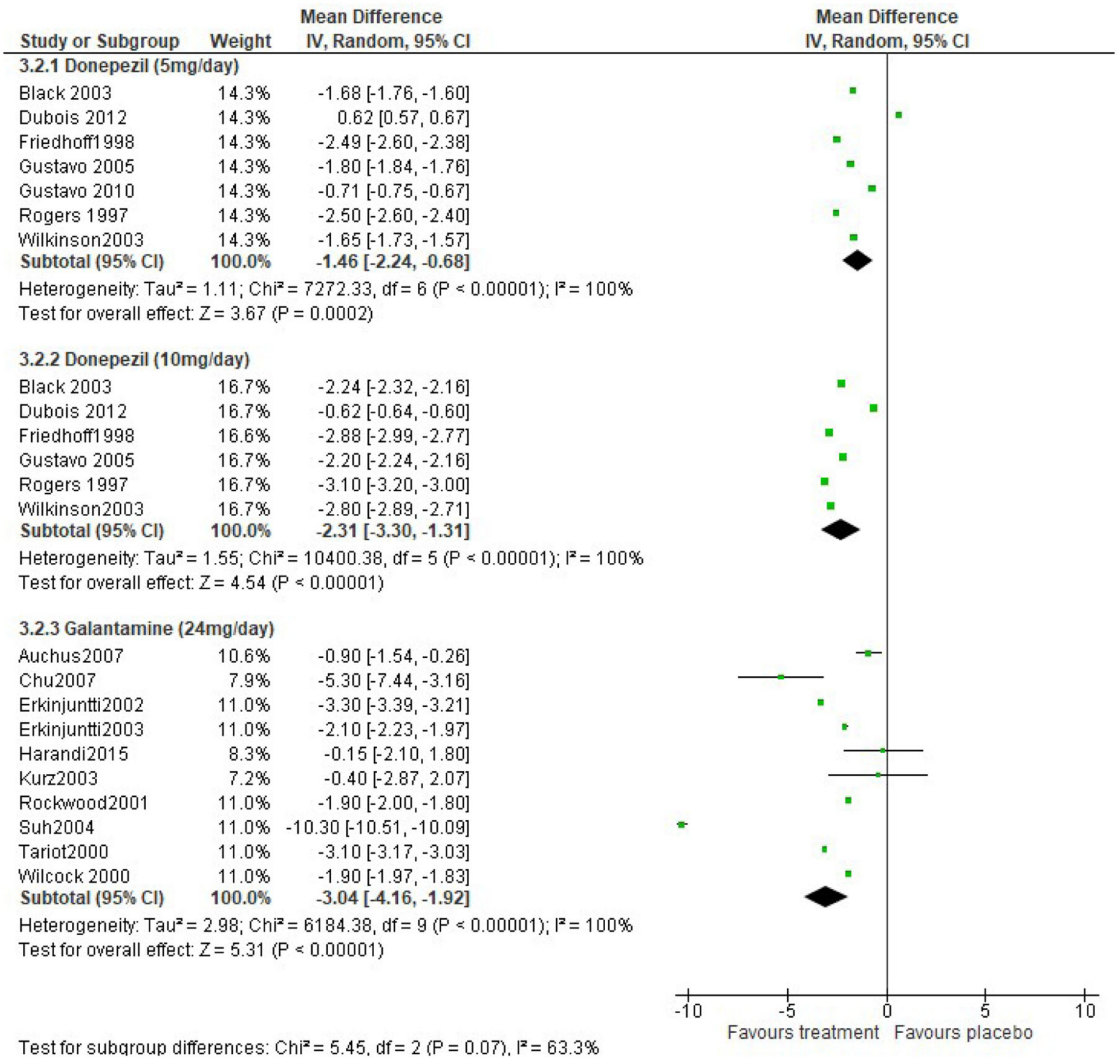
Forest plot of treatment effect of cholinesterase inhibitors on changes in cognitive symptom as measured by Alzheimer's disease assessment scale‐cognitive

Efficacy of donepezil 5 mg/day was reported by seven studies, donepezil 10 mg/day was reported by six studies and 10 studies reported efficacy data for galantamine 24 mg/day (Figure 3). There is evidence of heterogeneity in the combined difference of means for the efficacy of donepezil 5 mg/day (Cochran *Q* = 7272.33, *p* < 0.001, *I* ^2^ statistic = 100%), donepezil 10 mg/day (Cochran *Q* = 10400.38, *p* < 0.001, *I* ^2^ statistic = 100%) and galantamine (Cochran *Q* = 6184.38, *p* < 0.001, *I* ^2^ statistic = 100%). Patients treated with donepezil 5 mg/day, donepezil 10 mg/day, and galantamine 24 mg/day had improved ADAS‐cog score of −1.46 (95% CI = −2.24, −0.68, *z* = 3.67, *p* < 0.001, *α* = 0.05), −2.31 (95% CI = −3.30, −1.31, *z* = 5.45, *p* < 0.001, *α* = 0.05) and −3.04 (95% CI = −4.16, −1.92, *z* = 5.31, *p* < 0.001, *α* = 0.05) respectively. The pooled difference in the mean change from baseline Adas‐cog total score improved significantly with donepezil 5 mg/day, donepezil 10 mg/day, and galantamine 24 mg/day compared with placebo group.

### 3.4 Effectiveness of cholinesterase inhibitors to improve cognitive symptoms in people with dementia as measured by MMSE

**Figure 4:**
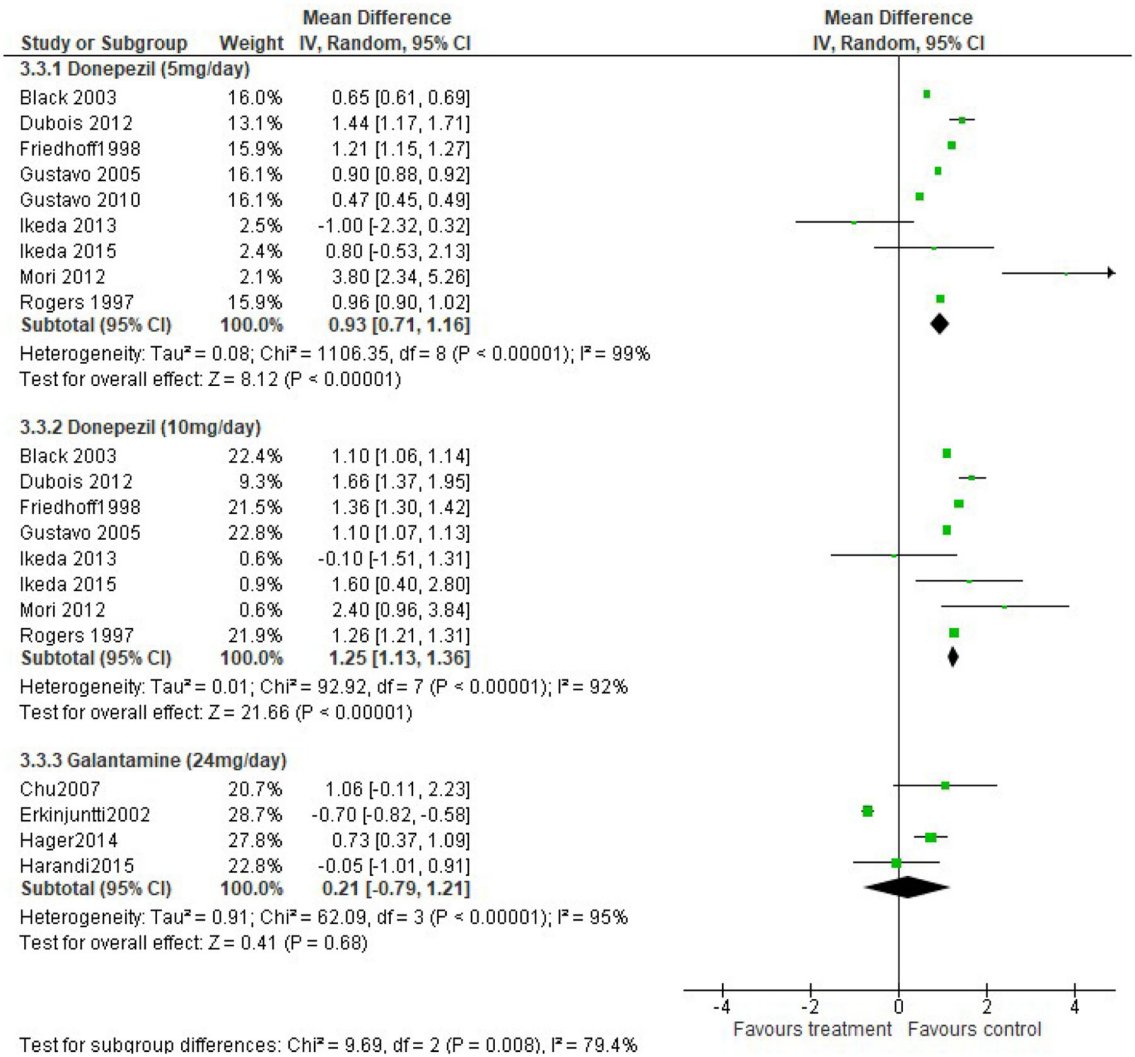
Forest plot of treatment effect of cholinesterase inhibitors on changes in cognitive symptom as measured by mini‐mental state examination at endpoint

Nine, eight, and four studies reported efficacy data for donepezil 5 mg/day, donepezil 10 mg/day, and galantamine 24 mg/day, respectively (Figure 4). Extreme heterogeneity was observed for the three cholinesterase inhibitors used (Cochran *Q* = 1106.35, *p* < 0.001, *I* ^2^ statistic = 99% for donepezil 5 mg/day; Cochran *Q* = 92.92, *p* < 0.001, *I* ^2^ statistic = 92% for donepezil 10 mg/day; Cochran *Q* = 62.09, *p* < 0.001, *I* ^2^ statistic = 95% for galantamine). Difference in mean change from baseline on MMSE score favored donepezil 5 mg/day, donepezil 10 mg/day but did not favor galantamine (SMD = 0.93, 95% CI = 0.71, 1.16, *z* = 8.12, *p* < 0.001, *α* = 0.05 for donepezil 5 mg/day; SMD = 1.25, 95% CI = 1.13, 1.36, *z* = 21.66, *p* < 0.001, *α* = 0.05 for donepezil 10 mg/day; SMD = 0.21, 95% CI = −0.79, 1.21, *z* = 0.41, *p* = 0.68, *α* = 0.05 for galantamine 24 mg/day).

## 4 DISCUSSION

### 4.1 Interpretation

This meta‐analysis evaluates the effect of cholinesterase inhibitors on cognitive and behavioral and psychological symptoms (BPSD) in people with dementia. Previous studies indicate that pharmacological interventions such as cholinesterase inhibitors are very effective in improving cognitive function and behavioral symptoms in people with dementia, however, there is the need to gather enough evidence for specific pharmacological interventions. In the current study, meta‐analysis indicated that cholinesterase inhibitors (donepezil 5 mg/day, donepezil 10 mg/day, and galantamine) are not effective in improving BPSD in people with probable dementia. Thus, the drugs or treatments (donepezil 5 mg/day, donepezil 10 mg/day) administered did not improve the NPI score in comparison to placebo or control group. This current meta‐analysis agrees with a recent study conducted by Raina et al.^37^ to evaluate the effect of donepezil (5 and 10 mg/day) on behavior/mood outcomes, using the NPI, showed no statistically significant changes relative to placebo. A meta‐analysis carried out by Dou et al.^6^ indicated that none of the cognitive enhancers evaluated was likely to improve behavioral symptoms. Thus, this confirms the findings of the current meta‐analysis. It is worthwhile that six studies evaluated the effect of galantamine 24 mg/day on BPSD. Four of the six studies demonstrated significant effects of galantamine 24 mg/day on BPSD, while only two studies fail to show an improvement on NPI score relative to placebo. However, the pooled or combined difference in the mean change of NPI score indicated that galantamine 24 mg/day is not statistically significantly effective in improving behavior symptoms in people with mild and probable dementia (MD = 0.11, 95% CI = −0.80, 1.02, *z* = 0.24, *p* = 0.81, *α* = 0.05).

The current meta‐analysis also suggests a significant benefit of cholinesterase inhibitors such as donepezil (5 and 10 mg/day) and galantamine relative to placebo on cognitive symptoms. Thus, the findings of the current study are in concordance with a previous meta‐analysis where 10 trials in 3239 people with mild to moderate dementia were reviewed to evaluate the effect of donepezil (5 and 10 mg/day) relative to placebo demonstrated consistent evidence of benefit in the domains of cognitive function on ADAS‐cog.^38^ Another researcher reviewed two large‐scales randomized trials for donepezil (10 mg/day) intervention where 1219 patients with probable or possible vascular cognitive impairment were enrolled and followed up for 24 weeks.^39^ Similar to the findings of the current study, the meta‐analysis they performed showed benefits associated with donepezil (10 mg/day) compared to placebo on cognitive function.

### 4.2 Strengths of the study

The use of meta‐analysis has improved the power of the effects of interest because of the increase in the sample size when a number of studies were combined. The use of meta‐analysis produces precise effects estimate and it is beneficial relative to systematic reviews. The findings from the meta‐analysis are more reliable than results of primary studies.

### 4.3 Limitations of the study

Even though we identified all relevant studies, there is an increased probability of overestimating the actual effect size because the meta‐analysis included only published studies. We did not apply Eagger's linear regression test to adjust for the observed bias.

## 5 CONCLUSION

The current meta‐analysis suggests a significant benefit of cholinesterase inhibitors such as donepezil (5 and 10 mg/day) and galantamine on cognitive symptoms. However, the combined difference in the mean change of NPI score indicated that donepezil (5 and 10 mg/day) and galantamine are not effective in improving BPSD in people with probable dementia. Based on the results of the current meta‐analysis, the food and drugs authority of various countries should consider the approval of donepezil (5 and 10 mg/day) and galantamine for the treatment of cognition symptoms of people with dementia.

## AUTHOR CONTRIBUTIONS

**Wisdom K. Takramah**: Conceptualization; data curation; formal analysis; investigation; methodology; project administration; resources; validation; visualization; writing – original draft; writing – review and editing. **Livingstone Asem**: Data curation; investigation; methodology; project administration; resources; writing – review and editing.

## CONFLICT OF INTEREST

The authors declare no conflict of interest.

## TRANSPARENCY STATEMENT

The lead author Wisdom Kwami Takramah affirms that this manuscript is an honest, accurate, and transparent account of the study being reported; that no important aspects of the study have been omitted; and that any discrepancies from the study as planned (and, if relevant, registered) have been explained.

## Data Availability

The authors confirm that the data supporting the findings of this study are available within the article [and/or] its supplementary materials.
